# Digital technologies to assess yoghurt quality traits and consumers acceptability

**DOI:** 10.1002/jsfa.11911

**Published:** 2022-05-10

**Authors:** Mitali K Gupta, Claudia Gonzalez Viejo, Sigfredo Fuentes, Damir D Torrico, Patrizia Camille Saturno, Sally L Gras, Frank R Dunshea, Jeremy J Cottrell

**Affiliations:** ^1^ School of Agriculture and Food, Faculty of Veterinary and Agricultural Sciences The University of Melbourne Parkville VIC Australia; ^2^ Future Food Hallmark Research Initiative The University of Melbourne Parkville VIC Australia; ^3^ Digital Agriculture, Food and Wine group The University of Melbourne Parkville VIC Australia; ^4^ Department of Wine, Food and Molecular Biosciences Lincoln University Lincoln New Zealand; ^5^ Philippine Carabao Center (PCC), National Headquarters and Gene Pool, Science City of Muñoz Palayan Philippines; ^6^ Department of Chemical Engineering and The Bio21 Molecular Science and Biotechnology Institute The University of Melbourne Parkville VIC Australia; ^7^ Faculty of Biological Sciences The University of Leeds Leeds UK

**Keywords:** biometrics, machine learning, physiological responses, emotions, near‐infrared

## Abstract

**BACKGROUND:**

Sensory biometrics provide advantages for consumer tasting by quantifying physiological changes and the emotional response from participants, removing variability associated with self‐reported responses. The present study aimed to measure consumers' emotional and physiological responses towards different commercial yoghurts, including dairy and plant‐based yoghurts. The physiochemical properties of these products were also measured and linked with consumer responses.

**RESULTS:**

Six samples (Control, Coconut, Soy, Berry, Cookies and Drinkable) were evaluated for overall liking by *n* = 62 consumers using a nine‐point hedonic scale. Videos from participants were recorded using the Bio‐Sensory application during tasting to assess emotions and heart rate. Physicochemical parameters Brix, pH, density, color (*L*, *a* and *b*), firmness and near‐infrared (NIR) spectroscopy were also measured. Principal component analysis and a correlation matrix were used to assess relationships between the measured parameters. Heart rate was positively related to firmness, yaw head movement and overall liking, which were further associated with the Cookies sample. Two machine learning regression models were developed using (i) NIR absorbance values as inputs to predict the physicochemical parameters (Model 1) and (ii) the outputs from Model 1 as inputs to predict consumers overall liking (Model 2). Both models presented very high accuracy (Model 1: *R* = 0.98; Model 2: *R* = 0.99).

**CONCLUSION:**

The presented methods were shown to be highly accurate and reliable with respect to their potential use by the industry to assess yoghurt quality traits and acceptability. © 2022 The Authors. *Journal of The Science of Food and Agriculture* published by John Wiley & Sons Ltd on behalf of Society of Chemical Industry.

## INTRODUCTION

Yoghurts are popular fermented products among consumers because of their flavor and health benefits.^1^ Yoghurt alternatives formulated from plant‐based sources have recently gained popularity because of growing vegetarianism and concerns over the environmental sustainability of traditional dairy products.^2^, ^3^ However, it has been reported that the inherent properties of the constituent proteins in plant‐based yoghurts will result in differences in gelation and texture compared to traditional dairy yoghurts.^4^ The sensory characteristics of a food product are highly affected by its physicochemical properties. Previous studies report that the sensory acceptance changes with flaxseeds^5^ or barley bran^6^ in a yoghurt formulation. Hence, it is important to understand the effect of both formulation and physicochemical properties on the sensory acceptability of yoghurt products.

The traditional method of sensory evaluation uses hedonic scales for self‐reporting liking levels of a product by consumers. However, this method can have some drawbacks because it lacks a measure of subconscious emotions expressed by the consumer.^7^ Emotions are important to measure, along with self‐reported liking, and help better understand the sensory attributes of food products.^8^ The advanced methods of understanding product liking by consumers involve using biometric technology to intrinsically measure the emotions and heart rate of the consumer, which relate to the acceptability of the product tasted. Heart rate variability is an objective method to evaluate physiological responses,^9^ which is usually measured by attaching electrodes to the body of an individual. A disadvantage is the awareness of the individual to analysis,^10^, ^11^ which can add a stressor to the response during evaluation.^12^ The non‐invasive method developed by Viejo *et al*.^10^ using computer vision analysis can overcome this limitation. This is achieved by capturing a video during the evaluation. The analysis focuses on the face's skin luminosity changes as a result of blood flow in the face using the green channel from the RGB color scale. Facial expression recognition has also been used to understand the subconscious responses from consumers towards a tasted food product.^13^, ^14^ Emotional response measurements may also provide more information on product liking during sensory evaluation.^15^ A combination of self‐reported, intrinsic facial expression recognition for emotional assessment and heart rate variability has been successfully implemented for the understanding of consumer acceptability of beer,^16^ chocolate,^17^, ^18^ insect‐based foods^19^ and coffee labels.^20^ Also, in yoghurt, a combination of sensory methods, including self‐reported and facial expression measurements,^21^ have been effectively used to understand consumers acceptability. Another study with yoghurts used augmented reality to predict liking for dairy and non‐dairy yoghurts, suggesting a strong association between liking and test environments. Hence, the use of augmented reality is a reliable alternative to predict consumer liking in different environments.^22^ The physicochemical attributes are important predictors of consumer liking and emotions, as studied for added cherry paste with sugar in a yoghurt,^23^ and a combination of aroma, color, texture and acidity affecting liking in another study for commercial Turkish yoghurts.^24^

Near‐infrared (NIR) spectroscopy, offering chemical fingerprinting of yoghurts at the 1596–2396 nm range, is a rapid, non‐invasive technique. It has the advantage of potentially being modelled to evaluate consumer liking of yoghurts without the requirement of carrying out testing with consumers after the process is standardized. NIR has also been previously modelled to assess the sensory and quality traits of beer^25^ and chocolate.^26^ The present study aimed to understand the effect of physicochemical properties of yoghurts on the emotional and physiological responses (biometrics) of consumers during sensory evaluation.

## MATERIALS AND METHODS

The present study measured physicochemical parameters, such as Brix, pH, density, color and firmness of yoghurt samples, along with the chemical fingerprinting of the yoghurts by measuring the NIR absorbance values. A consumer sensory session was conducted to assess overall liking and biometric responses, involving facial expressions to obtain emotional responses and heart rate as a physiological measure. Furthermore, two machine learning models were developed to predict (i) the seven physicochemical parameters using NIR absorbance values as inputs (Model 1) and (ii) overall liking of the samples using the physicochemical parameters obtained as outputs from Model 1 as inputs (Model 2).

### Samples

Six commercial yoghurt samples were used for all sensory and physiochemical tests. The samples were a combination of dairy and plant‐based yoghurts, with different consistency to measure the varying consumer responses to the different yoghurt types. These yoghurts were selected in a focus group study (*n* = 32), based on their preferences on taste, texture, appearance and emotions, as described previously,^27^ approved by the Human Ethics Committee at the University of Melbourne, Australia (Ethics ID 1545786.2 and 1853507.2). The sample products represent the following yoghurt types: control (plain dairy type), Coconut (plant‐based plain coconut type), Drinkable (sweetened dairy drinking type), Soy (plant‐based plain soy type), Cookies (sweetened dairy type with crunchies) and Berry (sweetened dairy type with berries).

### Yoghurt properties

#### Physicochemical measurements

A pH meter (Hanna Instruments Inc., Woonstock, RI, USA) was used to measure the pH of the yoghurts at ambient temperature (~25 °C). The device was previously calibrated with buffer solutions of pH 4.0 and pH 7.0. Furthermore, a handheld Brix meter (Alla‐France, Chemillé, France) was used to calculate the total soluble solids in °Bx. The density of the yoghurts was measured by dividing the weight of the samples by 50 mL of sample used. A Nix colorimeter (Nix Sensor Ltd, Hamilton, ON, Canada) was used to determine color indices for lightness (*L*), red/green (*a*) and yellow/blue (*b*) values for the yoghurt samples. The CIELab color scale indicates color index *L* from 0 to 100, measuring brightness from black to white. The color index *a* varies from a negative value (green) to positive (red), and color index *b* varies from a negative value (blue) to positive (yellow).^28^ All analyses were carried out in triplicates and averaged for analysis.

The firmness values were measured using a texture analyzer (TA.HD plus; Stable Microsystems, New Castle, DE, USA) with a 5‐kg load cell, using a trigger force of 1 g and a 10‐mm cylindrical probe.^29^ All measurements were performed at 10 °C, which was also the serving temperature for sensory analysis and analysis was performed in replicates of five.

#### NIR spectroscopy

The NIR absorbance values were measured within the 1596–2396 nm range using a microPHAZIR™ RX Analyzer (Thermo Fisher Scientific, Waltham, MA, USA). The samples were measured at room temperature (~25 °C) in triplicate and three measurements per replicate. Furthermore, the Savitzky–Golay first derivative was obtained for signal transformation to enhance peaks and plotting purposes using The Unscrambler X, version 10.3 (CAMO Software, Oslo, Norway).^25^

### Sensory evaluation and biometric measurements

A fully randomized consumer sensory session was conducted with 62 consumers. Power analysis was conducted using SAS Power and Sample Size, version 14.1 (SAS Institute, Cary, NC, USA) and confirmed that the number of participants was sufficient to find significant differences between samples (1 – β > 0.99). The yoghurt samples were labelled with three‐digit random codes and served at 10 ± 2 °C, as noted previously by Gupta *et al*.^21^ The ambient temperature of the tasting room was set to 22 ± 2 °C. All procedures were approved by the Human Ethics Advisory Group (HEAG) (Ethics ID 1853507.2). All participants provided their written informed consent form before participating in the yoghurt sensory sessions. Participants were chosen based on the frequency of consumption of yoghurts, and regular consumers of yoghurts who consumed yoghurt at least once per week were selected for the study. The participants included 67.7% females and 32.3% males, ranging from 21 to 58 years. The Bio‐Sensory app^30^ displayed the sensory questionnaire and recorded videos during the tasting. These videos were further used to analyze the emotional and physiological responses. The self‐reported liking for the yoghurts was assessed using a nine‐point hedonic scale (1: dislike extremely; 5: neither like nor dislike; 9: like extremely).

Heart rate (HR) was analyzed from the recorded videos via a customized Matlab® R2021a (Mathworks, Inc., Natick, MA, USA) algorithm using the green channel from the RGB color code as explained by Viejo *et al*.^10^ The selected region of interest was the forehead to estimate HR in beats per minute because it is one of the areas with the highest blood flow in the face. The videos were also used to analyze the facial expressions and emotional responses of participants. These videos were analyzed from when the sample was first put in the mouth to assess the first reaction of the consumer. Facial muscles and head movements from the participants when evaluating the samples were recorded and analyzed using a facial expression recognition computer application developed by the Digital Agriculture Food and Wine group (DAFW) from the University of Melbourne (UoM), based on the *Affectiva* software development kit (SDK; *Affectiva*, Boston, MA, USA). The software can track the human face based on the Viola–Jones cascade detector algorithm and can detect the micro‐ and micromovements using the histogram of the oriented gradient algorithm and with support vector machine (SVM) machine learning method, to automatically translate facial expressions into specific emotions and emojis.^31^, ^32^ In total, 45 parameters were generated from the software, grouped as intensity (2), facial expression (21), head orientation (3), emotions (7) and emoji (12) (Table 1).^19^

**Table 1:** Facial expressions and emotion parameters obtained from the facial expression recognition software (*Affectiva*)

Category	Parameter		Label	Category	Parameter		Label
*Intensity*	*1*	*Valence*	Valence	*Head Orientation*	*24*	*Pitch*	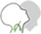
	*2*	*Engagement*	Engagement		*25*	*Yaw*	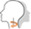
*Facial Expression*	*3*	*Smile*	Smile		*26*	*Roll*	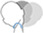
	*4*	*Inner Brow Raise*	Inner Brow Raise	*Emotion*	*27*	*Joy*	Joy
	*5*	*Brow Raise*	Brow Raise		*28*	*Surprise*	Surprise
	*6*	*Brow Furrow*	Brow Furrow		*29*	*Fear*	Fear
	*7*	*Nose Wrinkle*	Nose Wrinkle		*30*	*Disgust*	Disgust
	*8*	*Upper Lip Raise*	Upper Lip Raise		*31*	*Sadness*	Sadness
	*9*	*Lip Corner Depressor*	Lip Corner Depressor		*32*	*Anger*	Anger
	*10*	*Chin Raise*	Chin Raise		*33*	*Contempt*	Contempt
	*11*	*Lip Pucker*	Lip Pucker	*Emoji*	*34*	*Relaxed*	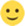
	*12*	*Lip Press*	Lip Press		*35*	*Smiley*	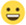
	*13*	*Lip Suck*	Lip Suck		*36*	*Laughing*	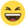
	*14*	*Mouth Open*	Mouth Open		*37*	*Kissing*	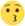
	*15*	*Smirk*	Smirk		*38*	*Wink*	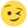
	*16*	*Eye Closure*	Eye Closure		*39*	*Stuck Out Tongue Winking Eye*	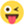
	*17*	*Attention*	Attention		*40*	*Stuck Out Tongue*	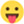
	*18*	*Eye Widen*	Eye Widen		*41*	*Disappointed*	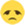
	*19*	*Cheek Raise*	Cheek Raise		*42*	*Rage*	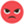
	*20*	*Lid Tighten*	Lid Tighten		*43*	*Smirk*	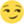
	*21*	*Dimpler*	Dimpler		*44*	*Flushed*	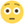
	*22*	*Lip Stretch*	Lip Stretch		*45*	*Scream*	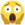
	*23*	*Jaw Drop*	Jaw Drop				

The underlined terms were used for statistical analysis in the present study.

### Statistical analysis and machine learning modelling

An analysis of variance (ANOVA) was conducted to assess significant differences (*P* < 0.05) between samples for the overall liking scores, heart rate, emotional responses and physicochemical parameters using Fisher's least significant difference (LSD) post‐hoc test (α = 0.05). This was performed using Minitab® Statistical, version 19.1.1 (Minitab Inc., State College, PA, USA). The Matlab® R2021a was also used to plot the NIR curves and first derivative. A multivariate data analysis based on principal component analysis (PCA) was conducted to assess relationships and associations among samples and variables from the biometrics and physicochemical parameters. Furthermore, a matrix was developed to find significant correlations (*P* < 0.05) between all parameters.

**Figure 1:**
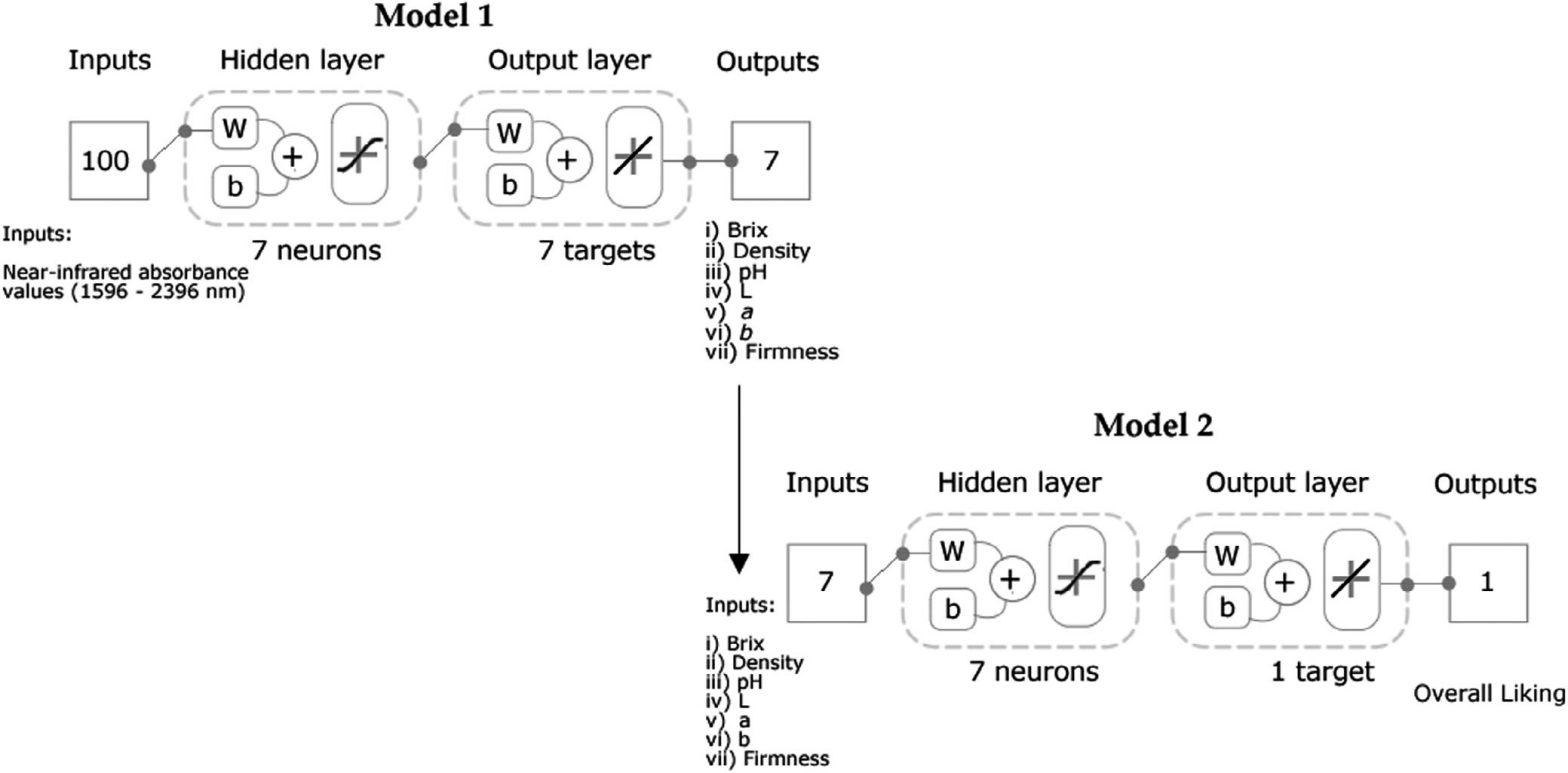
Diagrams of the feedforward artificial neural network models. Models consist of a hidden layer with a tan‐sigmoid function and an output layer with a linear transfer function. Abbreviations: W, weights; b, bias.

Two machine learning regression models based on artificial neural networks were developed using a customized code written in Matlab® R2021a by the DAFW‐UoM. Seventeen training algorithms were tested in a loop to find the best model based on the correlation coefficient (*R*) and performance assessed with the mean squared error (MSE). The Bayesian Regularization algorithm produced the best models with no overfitting; this algorithm performs very well with noisy and/or small datasets.^33^ Model 1 was developed using the 100 NIR absorbance values (1596–2396 nm) as inputs to predict the physicochemical parameters: (i) Brix, (ii) density, (iii) pH, (iv) L, (v) *a*, (vi) *b* and (vii) firmness. The device used to measure NIR provides values for wavelengths every 7–9 nm. On the other hand, Model 2 was constructed using the seven physicochemical parameters to predict the overall liking of the yoghurt samples (Fig. 1). Both models were developed using a random data division with 65% of the samples (*n* = 35; observations = 245) used for training and 35% of the samples (*n* = 19; observations = 133) for testing. A neuron trimming test was performed using three, five, seven and ten neurons, with seven resulting in the best with no under or overfitting of the models.

## RESULTS AND DISCUSSION

### Physicochemical characteristics of yoghurts

**Figure 2:**
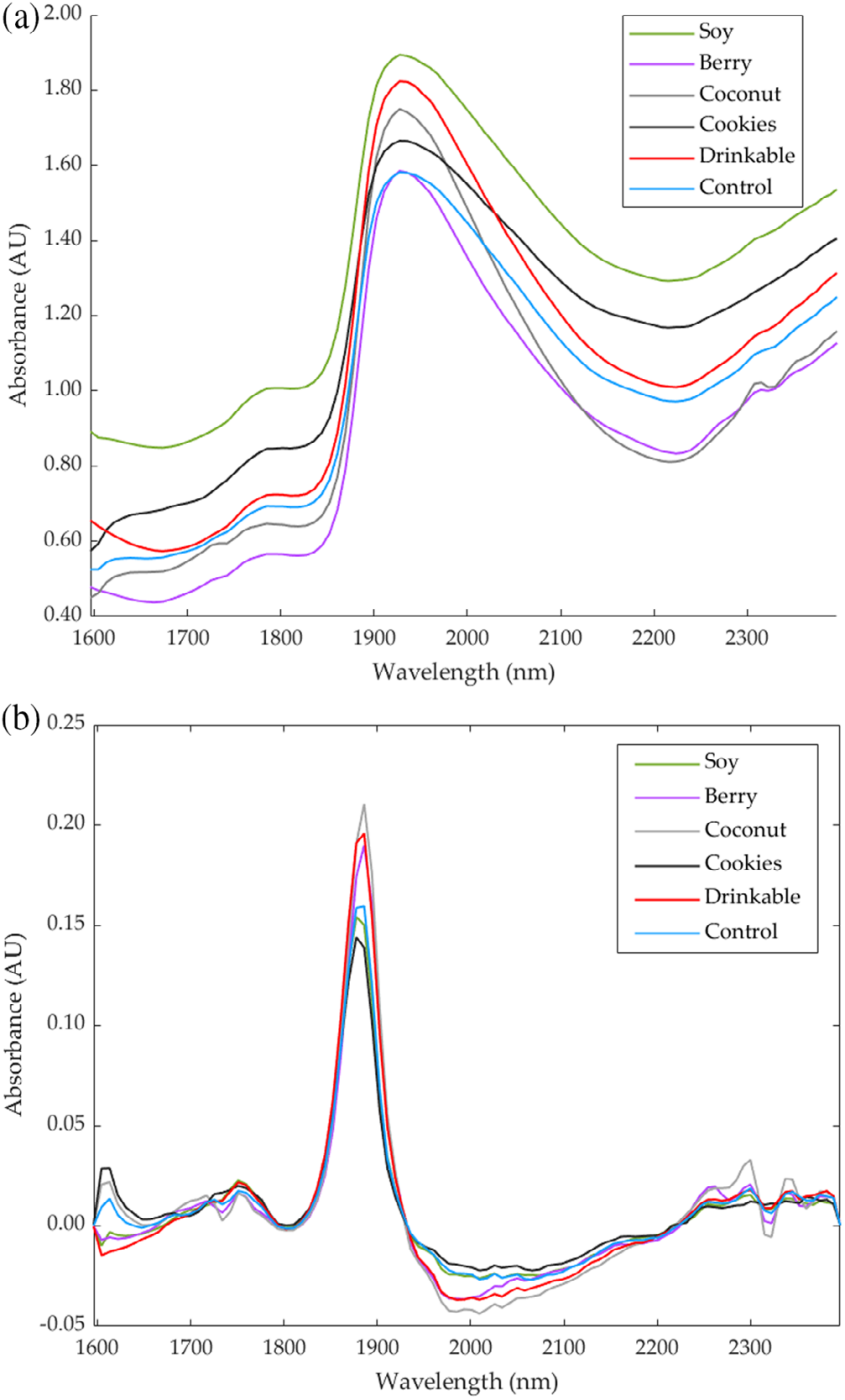
Near‐infrared curves showing (a) the raw and (b) first derivative of absorbance within the range 1597–2400 nm.

The NIR raw absorbance of the six yoghurts is shown in Fig. 2(a). The major peak occurred between 1900 and 2000 nm for all samples, with the highest absorbance occurring for Soy yoghurt and lowest absorbance for Berry yoghurt. The overtone for water, a major constituent of yoghurt, has previously been identified at 1940 nm,^34^ consistent with this major peak. The absorption bands for lactic acid occur between 1800 and 2000 nm^35^ but are not visible as a result of the overlapping broad water peak.

The first derivative of the NIR absorbance is shown in Fig. 2(b) to allow greater observation of peaks between 1750 and 1800 nm, 1900 and 1950 nm, 1980 and 2050, and 2250 and 2350 nm. Peaks between 1980 and 2050 nm and between 2180 and 2470 nm, identified as overtones for proteins,^34^, ^36^ were observed in all samples, although other components can also contribute to the higher wavelength range. The Cookies yoghurt had peaks with the highest absorbance between 1980 and 2050 nm and the Coconut yoghurt the lowest absorbance in this range, consistent with the different formulations, including the higher protein content of the Cookie yoghurt compared to the Coconut yoghurt [9.7% (w/w) and 0.7% (w/w) protein, respectively]. Aromatic compounds may also have contributed to absorbance between 1980 and 2050 nm.^34^

Table 2 shows the Brix, pH, density, firmness and color values for the yoghurt samples. There were significant (*P* < 0.05) differences between samples for all parameters. The lowest pH value was observed for Cookies yoghurt at 4.21, and the highest value was observed for Soy (4.76) and Berry (4.74) yoghurts. A previous study has shown that an increase in pH value strengthens the buttery, creamy and sweet after‐taste in yoghurts for a given formulation^37^; hence, it is an important parameter to evaluate sensory differences among yoghurts. The pH values in the present study are comparable to previous research.^38^

**Table 2:** Mean values of the physicochemical characteristics of each yoghurt sample

Parameter	Control	Coconut	Drinkable	Soy	Cookies	Berry
Color						
*L*	74.7^ab^ ± 2.50	71.6^b^ ± 2.32	76.1^a^ ± 0.91	74.0^ab^ ± 0.10	75.8^a^ ± 0.14	64.1^c^ ± 1.47
*a*	1.36^bc^ ± 0.10	0.28^c^ ± 0.11	2.08^bc^ ± 0.05	2.44^b^ ± 2.12	0.54^bc^ ± 0.05	4.64^a^ ± 0.53
*b*	8.68^ab^ ± 0.10	3.92^b^ ± 0.11	8.86^ab^ ± 0.05	26.5^a^ ± 2.12	9.08^ab^ ± 0.05	1.02^b^ ± 0.53
Brix (°Bx)	10.5^d^ ± 0.29	8.00^e^ ± 0.29	12.0^c^ ± 0.29	6.50^f^ ± 0.29	13.7^b^ ± 0.33	21.33^a^ ± 0.88
pH	4.44^c^ ± 0.01	4.25^d^ ± 0.01	4.53^b^ ± 0.01	4.76^a^ ± 0.00	4.21^e^ ± 0.00	4.74^a^ ± 0.02
Density (g mL^1^)	1.04^bc^ ± 0.01	1.03^bc^ ± 0.02	1.04^b^ ± 0.00	0.98^d^ ± 0.01	1.01^c^ ± 0.00	1.08^a^ ± 0.01
Gel firmness (N)	0.10^d^ ± 0.02	0.15^b^ ± 0.00	0.02^e^ ± 0.00	0.13^c^ ± 0.00	0.32^a^ ± 0.00	0.09^d^ ± 0.00

Values represent the mean ± SE (*n* = 3). Different lowercase letters within a row denote significant differences between samples based on ANOVA and Fisher's LSD post‐hoc test at *P* < 0.05.

The total soluble solids, indicated by the Brix value, was highest for Berry (21.3 °Bx) and lowest for Coconut yoghurt (8.00 °Bx), showing significant differences (*P* < 0.05) between the samples. Brix is considered the best objective measurement for sugar content in a food product. In an evaluation with apples, it was observed that a difference of more than 1 °Bx is required for a perceivable sweetness difference.^39^ This explains the higher sugar content in Berry, and hence sweetness, compared to other samples.

Moreover, the Berry sample (1.08 g mL^−1^) had the highest value for density compared to the other products, and the lowest was observed for Soy (0.98 g mL^−1^). The firmness values (Table 2) were highest for Cookies (0.32 N), followed by Coconut (0.15 N), and lowest for Drinkable (0.02 N) yoghurt. The Cookies yoghurt had the highest protein content, which may lead to an increase in firmness.^40^ The presence of fat and hydrocolloids, both being present in the Coconut yoghurt, which has lower protein, can also affect firmness.^41^ By contrast, the shearing action performed in the formation of the Drinkable yoghurt leads to a lower firmness.^42^

The highest *L* color index values were displayed by the Cookies (75.82) and Drinkable (76.10) yoghurts, whereas Berry (64.10) showed the lowest value. The lightness of yoghurts is associated with the particle size of fats and proteins and is also influenced by processing parameters, such as stirring and homogenization.^43^ The color index *a* was highest for Berry (4.64) and lowest for Coconut (0.28). Additionally, color index *b* was highest for Soy (26.46) and lowest for Coconut (3.92) and Berry (1.02), which is in agreement with the results reported by Grasso *et al*.^41^ Overall liking has been observed to significantly relate to color for dairy products, like cheese^44^; hence, color measurements are important in understanding consumer acceptability.

### Sensory characteristics of yoghurts

#### Self‐reported liking

**Figure 3:**
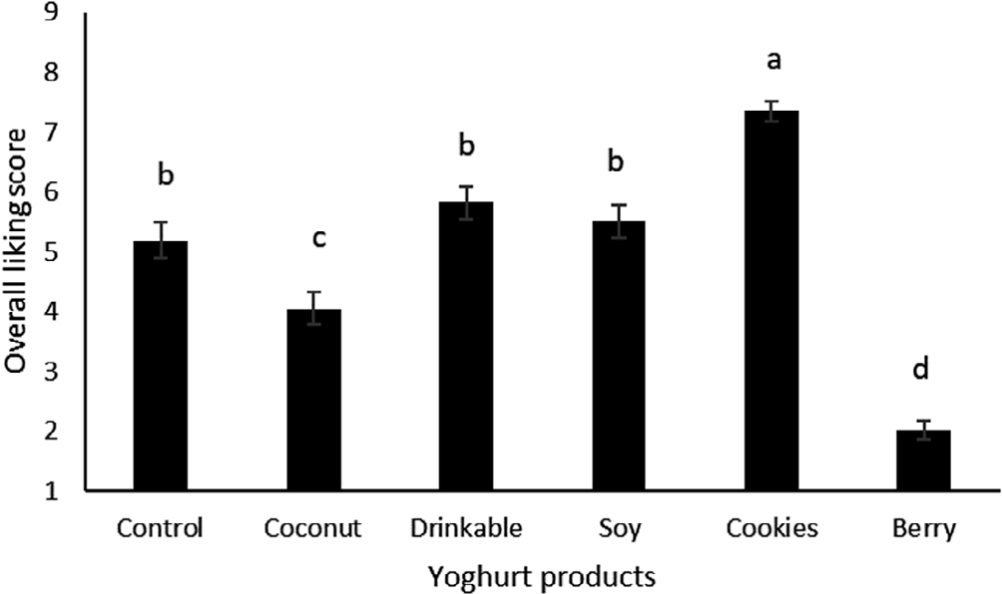
Self‐reported Overall liking scores for each yoghurt sample on a nine‐point hedonic scale. Values represent the means of 62 participants, and error bars represent the SE based on ANOVA and Fisher's LSD post‐hoc test at *P* < 0.05. Superscripts on each bar represent significant differences.

The overall liking scores self‐reported by consumers on a nine‐point hedonic scale (Fig. 3) showed that there were significant differences between samples (*P* < 0.05). Cookies (7.36) was the most liked yoghurt product, whereas Berry (2.01) was the least liked. Cookies yoghurt has cereal particles. This combination has been associated with additional health benefits,^45^ and hence may contribute to increasing its liking scores, as seen in the present study. Because of increasing health consciousness, consumers are moving away from high sugar foods.^46^ This may explain the lower liking of the Berry yoghurt, which showed the highest value of Brix. Another factor that can influence liking is the product firmness. Cookies yoghurt showed the highest firmness values, and also the highest overall liking scores. Previous studies have shown yoghurt firmness to be positively linked to protein content, hence enhancing yoghurt liking.^27^ Control, Soy and Drinkable samples were similarly liked, followed by Coconut. Previous studies have also shown that dairy and soy yoghurts were similarly liked by consumers.^41^ Consumers like sweet products, but too high a sweetness is disliked and reduces liking, as observed in the case of Berry yoghurt. There have been studies on reducing sugar content in yoghurts as a result of increasing health needs.^47^ Previous reports suggest that consumers prefer a medium level of sweetness in yoghurts.^48^

#### Biometric responses

Table 3 shows significant differences (*P* < 0.05) for emotional responses, including lip press, 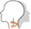 (yaw), Surprise and heart rate. Lip press was highest for Drinkable (10.18) and lowest for Soy (5.42) and Berry (4.36) samples. 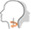 (yaw) was highest for Cookies (−0.59) and lowest for Berry (−6.20), whereas Surprise was the highest for Coconut (2.85). Cookies (82.16 BMP) presented the highest value for heart rate, whereas Berry (74.79 BMP) displayed the lowest value. Facial expressions and physiological responses, such as heart rate, are important parameters for understanding consumer liking and have been successfully used to associate liking towards breakfast drinks.^49^ An increased heart rate is also associated with a higher liking for tasted food products. Similar results were observed in the present study, where Berry, the least‐liked sample, decreased the heart rate of participants. However, in other studies, such as for chocolate^17^ and beer^16^ tasting, the heart rate did not indicate significant differences between the sample types tested. This shows that the physiological responses of consumers depend on the food stimuli to which they are exposed.

**Table 3:** Overall mean values for the facial expression recognition responses of yoghurt samples that are significantly different

Type	Parameter	Control	Coconut	Drinkable	Soy	Cookies	Berry
*Facial Expression*	*Facial expression*	7.90^ab^ ± 1.64	6.77^ab^ ± 1.21	10.2^a^ ± 1.74	5.42^b^ ± 1.02	7.06^ab^ ± 1.24	4.36^b^ ± 0.86
	Lip Press						
	*Head Orientation*						
	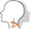 (Yaw)	−3.48^abc^ ± 1.39	−4.88^bc^ ± 1.28	−4.02^abc^ ± 1.56	−1.61^ab^ ± 1.24	−0.59^a^ ± 1.36	−6.20^c^ ± 1.07
	*Emotion*						
	Surprise	1.15^b^ ± 0.34	2.85^a^ ± 0.92	0.82^b^ ± 0.22	1.02^b^ ± 0.30	0.85^b^ ± 0.17	1.32^b^ ± 0.34
*Heart rate*	Heart rate (BPM)	77.1^ab^ ± 2.23	77.4^ab^ ± 2.39	78.3^ab^ ± 2.84	75.6^ab^ ± 1.94	82.2^a^ ± 2.30	74.8^b^ ± 2.28

Values represent the mean ± SE (*n* = 62). Different lowercase letters within a row denote significant differences between samples based on ANOVA and Fisher's LSD post‐hoc test at *P* < 0.05.

### Multivariate data analysis

**Figure 4:**
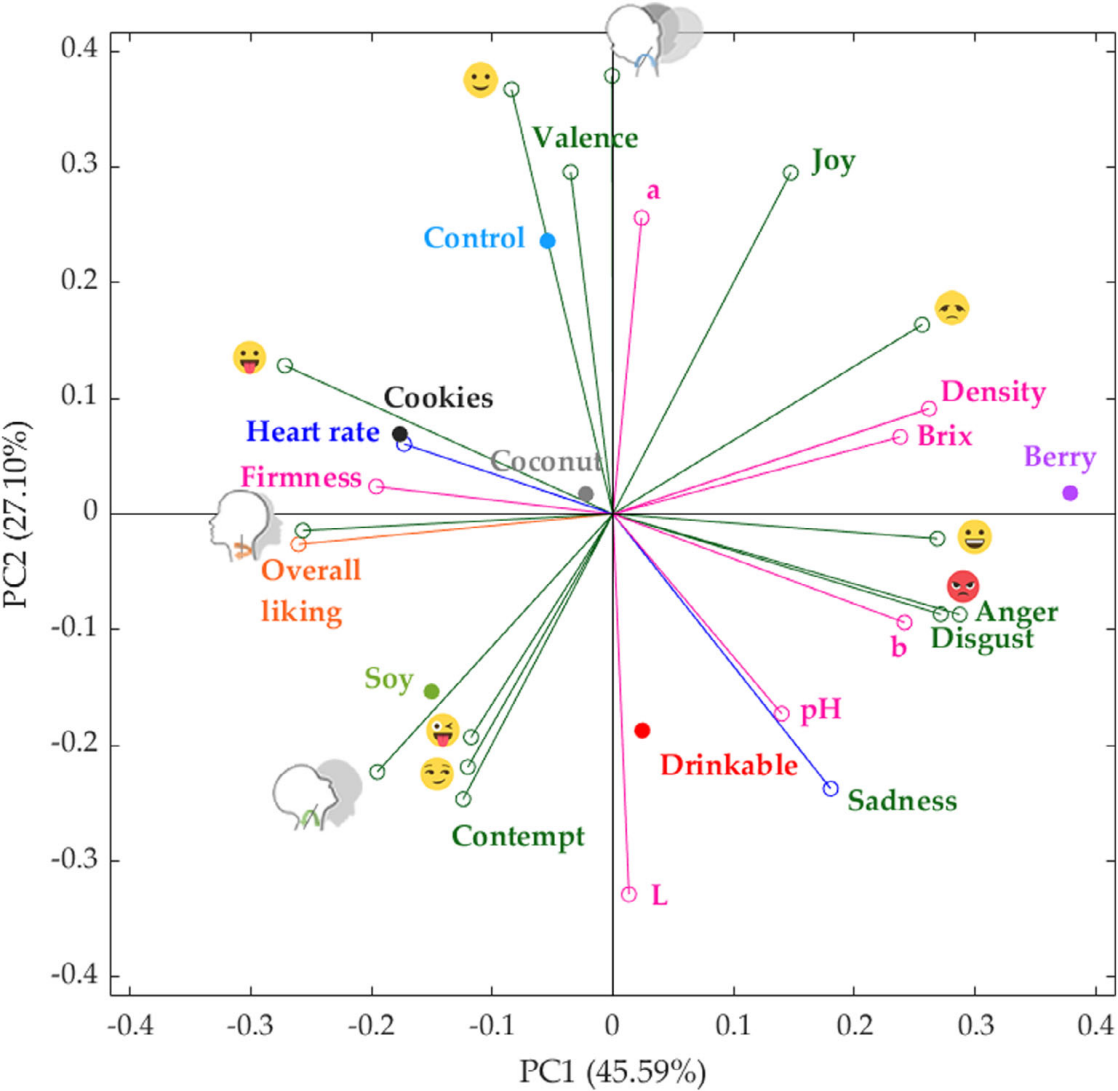
Principal components analysis for the self‐reported Overall liking, emotional and physiological responses related with the physicochemical parameters of the six yoghurt products tasted by consumers. PC1 and PC2 refer to principal components one and two, respectively.

PCA explained 72.69% of the total data variability (PC1 = 45.59%, PC2 = 27.10%) (Fig. 4). Principal component one (PC1) was mainly represented by overall liking (−0.26), firmness (−0.20), heart rate (−0.17), 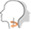 (yaw; −0.26) and 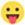 (stuck out tongue; −0.27) on the negative side, and represented brix (0.24), density (0.26), color index *b* (0.24), anger (0.29), disgust (0.27), 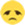 (disappointed; 0.26), 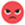 (rage; 0.29) and 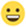 (smiley; 0.27) on the positive side. Principal component two (PC2) was mainly linked to valence (0.30), color index *a* (0.26), joy (0.29), 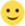 (relaxed; 0.37) and 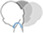 (roll; 0.38) on the positive side, whereas it was mainly linked to pH (−0.17), color index *L* (−0.33), sadness (−0.24), contempt (−0.25), 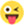 (stuck out tongue with winking eye; −0.19), 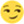 (smirk; −0.22) and 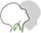 (pitch; −0.22) on the negative side.

Overall liking was directly linked to heart rate, 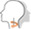 (yaw) and firmness, which is confirmed with the highest heart rate (Table 3), firmness values (Table 2) and overall liking scores (Fig. 3) for Cookies yoghurt. By contrast, overall liking was negatively linked to Brix and density, which shows a relationship with Berry yoghurt. Cookies and Coconut samples were associated with heart rate, firmness, overall liking, 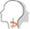 (yaw), and 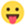, whereas the Soy sample was associated with overall liking, 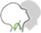 (pitch), 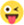 (stuck out tongue with winking eye), 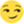 (smirk) and Contempt. Control was mainly related to 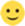 (relaxed), Valence, 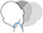 (roll) and color index *a*. On the other hand, Berry yoghurt was associated with density, Brix, 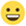 (smiley), 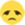 (disappointed) and 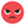 (rage). Drinkable sample was linked with pH, color index *L* and sadness.

The PCA had a combination of dairy and plant‐based yoghurts distributed on the positive and negative axis, which shows that the protein base was not the only deciding factor for consumer acceptability of yoghurts. Key physicochemical parameters (pH, Brix, density, firmness and color indices) that induced different biometric responses appeared to main contributors to yoghurt acceptability among the consumers. The Cookies, Coconut and Soy samples were directly associated with overall liking, whereas Berry was inversely related with overall liking. In previous studies, an implicit approach found more intense negative emotions displayed for disliked juice samples.^50^ A similar response was observed in the present study, with Berry being the least liked yoghurt, separated from the other examined products.

**Figure 5:**
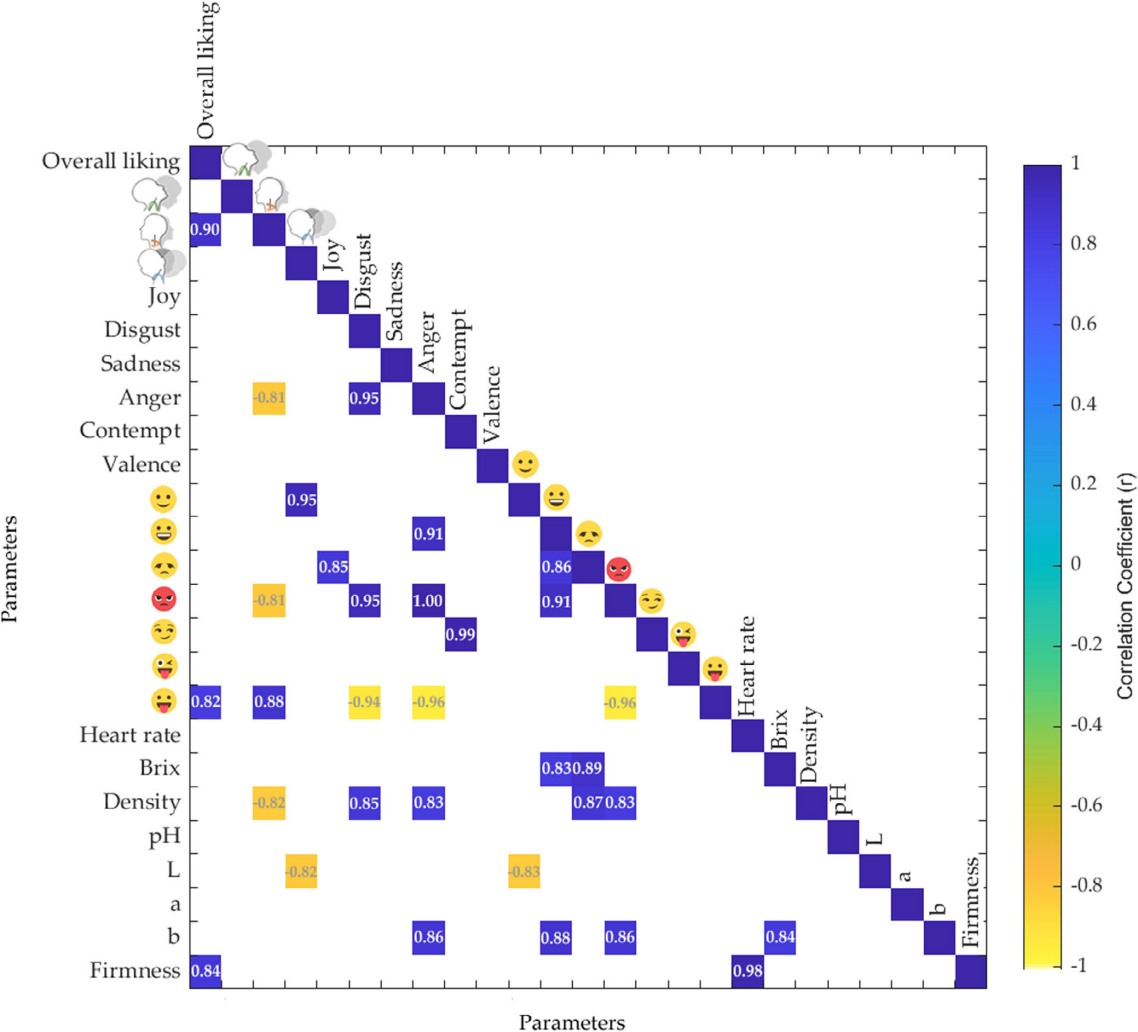
Matrix showing significant (*P* < 0.05) correlations between the self‐reported overall liking, emotional and physiological responses with the physicochemical parameters of the yoghurts. Color bar: blue side shows the positive, whereas the yellow side represents the negatives correlations. Darker colors indicate the highest or lowest correlations.

A matrix was also plotted to represent the significant correlations between the sensory descriptors and the physicochemical parameters of the yoghurts (Fig. 5). It was observed that the self‐reported overall liking score was positively correlated with 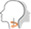 (yaw; *r* = 0.90), 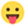 (stuck out tongue; *r* = 0.82) and firmness (*r* = 0.84). This is in accordance with a previous study, which reported that consumers have a higher perceived liking towards a yoghurt with a firm and dense texture.^51^ Heart rate was also positively correlated with firmness (*r* = 0.98). Brix had a positive correlation with 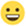 (smiley; *r* = 0.83), 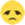 (disappointed; *r* = 0.89) and *b* color index (*r* = 0.84). Density showed a positive correlation with disgust (*r* = 0.85), anger (*r* = 0.83), 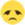 (disappointed; *r* = 0.87) and 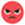 (rage; *r* = 0.83), whereas, negatively correlated with 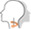 (yaw; *r* = −0.82). The *L* color index was found to be negatively correlated with 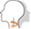 (yaw; *r* = −0.82) and 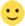 (relaxed; *r* = −0.83), whereas, the *b* color index was positively correlated with anger (*r* = 0.86), 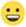 (smiley; *r* = 0.88) and 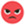 (rage; *r* = 0.86). However, the pH and *a* color index did not significantly correlate with any of the other parameters. The pH of yogurt is known to correlate with the perceived acidity of yoghurt.^37^ In a previous study, a strong influence of pH was observed on the flavor attributes perceived in yoghurt, with a difference of 0.4 pH units being sufficient to detect a sensory difference.^37^ However, in the present study, the pH did not influence acceptability, even with a maximum pH difference of 0.55 between the samples (Table 2).

Overall, the matrix showed that the physicochemical attributes of yoghurts, including density, Brix and *b* color index, were positively correlated with negative emotions. However, none of these physicochemical parameters and heart rate were significantly correlated with the self‐reported Overall liking. The head orientation 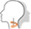 (yaw) was correlated with positive terms, showing that it was a factor indicating likability, meaning that consumers tend to rotate their heads to the right when they like a product. This coincides with previous studies reporting that, with highly bitter food, both adults and babies tend to rotate the head to the left as a sign of rejection towards potentially toxic substances.^16^, ^52^, ^53^ The emoji 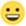 (smiley) did not appear to be an effective discriminator for yoghurt products because it correlated with negative rather than positive terms, suggesting misclassification. This shows that a combination of biometrics and self‐reported liking can provide more extensive details about consumer acceptability towards yoghurts.

### Machine learning modelling

**Figure 6:**
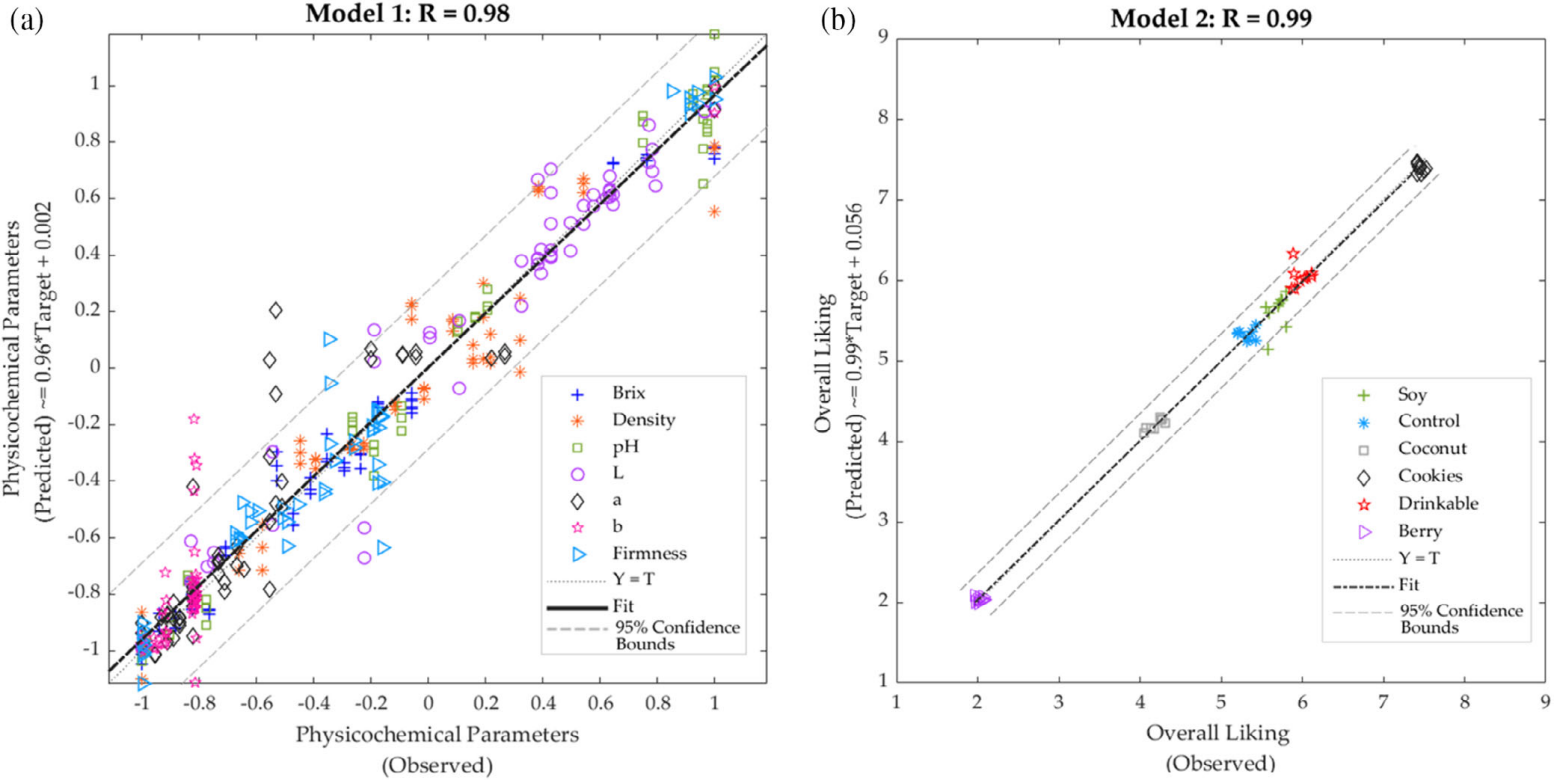
Overall artificial neural network models developed using (a) the near‐infrared absorbance values to predict physicochemical parameters of yoghurt (Model 1) and (b) the physicochemical parameters of yoghurts to predict consumers overall liking (Model 2).

Table 4 shows that Model 1 had very high overall accuracy (*R* = 0.98) to predict seven physicochemical parameters of yoghurts using NIR absorbance values as inputs with a slope close to the unity (*b* = 0.96). The model does not show any sign of under or overfitting because the accuracies of the three stages are all > 0.95, and the training performance value (MSE = 0.01) is lower than the testing value (MSE = 0.04). Likewise, Model 2 had very high accuracy (*R* = 0.99) using the outputs from Model 1 (physicochemical parameters) as inputs to predict the overall liking of the yoghurt samples. This model had very high slope values (*b* > 0.98) for the three stages and no signs of under or overfitting because the three stages had the same accuracy, and the training performance value (MSE < 0.01) was lower than the testing value (MSE = 0.04).Figure 6(a) shows the overall Model 1 with 5% outliers (19 out of 378 data points) comprising most of these the color parameters (16 outliers). This may be a result of the spectral range, which is above the UV‐visible color spectra, which may not represent as accurately visible colors. However, most of the data points from color are within the 95% confidence bounds, which are a result of proteins such as casein and fat that are responsible for the white color and lightness of milk in the case of dairy products^54^ and associated with anthocyanin content in soybeans varieties, giving a yellow color in soy yoghurt.^55^ Furthermore, most of the outliers in Model 1 were from the drinkable yoghurt, with one from soy samples. On the other hand, Fig. 6(b) shows that there were 5.5% outliers (three out of 54 data points), one outlier from the Drinkable yoghurt and two from the Soy samples for Model 2. As can be observed, in both Models, the Drinkable and Soy yoghurts were responsible for the outliers. These samples can also be observed in the NIR in Fig. 2 (a), in which Soy and Drinkable samples had the highest absorbance values. Furthermore, Soy and Drinkable yoghurts are also observed in the PCA (Fig. 4), grouped in the negative side of PC2, compared to the other samples located on the positive side of PC2.

**Table 4:** Statistical results of the artificial neural network models showing the correlation coefficient (*R*), slope (*b*) and performance based on mean squared error (MSE) for each stage

Stage	Samples	Observations	*R*	Slope (*b*)	Performance MSE
Model 1: Inputs: Near‐infrared absorbance values; Targets: Physicochemical parameters					
Training	35	245	0.99	0.97	0.01
Testing	19	133	0.95	0.95	0.04
Overall	54	378	0.98	0.96	–
Model 2: Inputs: Physicochemical parameters; Targets: Overall liking					
Training	35	35	0.99	1.00	< 0.01
Testing	19	19	0.99	0.98	0.04
Overall	54	54	0.99	0.99	–

These models may potentially be used in the food industry to predict physicochemical parameters as yoghurt quality traits and overall liking by measuring yoghurt samples using only NIR readings. However, in the case of small companies that may not have sufficient budget to acquire a NIR device, Model 2 can be used to predict overall liking by measuring the seven simple physicochemical parameters. A similar approach was presented by Gunaratne *et al*.^26^ for chocolate and by Viejo *et al*.^56^ for beer samples produced using sonication; in both studies, the artificial neural network models had very high accuracies (*R* > 0.94), similar to the high accuracy reported in the present study.

## CONCLUSIONS

Similarities were observed between the plain dairy and plain plant‐based yoghurt types, also generating similar liking. The study further establishes the link between the physicochemical and sensory parameters, especially between the dairy yoghurts and the plant‐based alternatives, as well as between the different yoghurt types. The association of facial expressions with heart rate and liking improved understanding of the consumers' acceptability towards yoghurts by evaluating the most‐ and the least‐liked formulations. The advantage of using NIR for the chemical fingerprinting of yoghurts has also been established in the present study as a rapid, simple and non‐invasive method to predict consumer liking of yoghurt. The potential use of these models for the industry is to assess a large number of samples within the production process, hence making the product development process much faster. These models can further be used to predict the yoghurt quality traits and consumers acceptability in a more efficient and less time‐consuming way. These emotional and physiological responses can be tested for more plant‐based products to further understand the effect of protein bases in future studies. The relationship of sugar concentration to the liking of yoghurts can be tested to establish the maximum concentration appreciated by consumers in flavored yoghurt. Furthermore, other product parameters, including rheology, can also be used to develop machine learning models to predict sensory attributes of yoghurt products.
